# Increasing Screening Intentions for Oral and Pharyngeal Cancer

**DOI:** 10.1007/s12160-013-9480-z

**Published:** 2013-03-12

**Authors:** Henrietta L. Logan, James A. Shepperd, Elizabeth Pomery, Yi Guo, Keith E. Muller, Virginia J. Dodd, Joseph L. Riley

**Affiliations:** 1 Department of Community Dentistry and Behavioral Science, Southeast Center for Research to Reduce Disparities in Oral Health, University of Florida, Gainesville, FL 32610-32608 USA; 2 Department of Psychology, Southeast Center for Research to Reduce Disparities in Oral Health, University of Florida, Gainesville, FL 32611-2250 USA; 3 Southeast Center for Research to Reduce Disparities in Oral Health, University of Florida, Gainesville, FL 32610-3628 USA; 4 Department of Health Outcomes and Policy, Southeast Center for Research to Reduce Disparities in Oral Health, University of Florida, Gainesville, FL 32610-3628 USA

**Keywords:** Oral and pharyngeal cancer, Media campaign, Rural health, Health disparities, Intentions

## Abstract

**Background:**

Oral and pharyngeal cancer is a serious health threat that goes unnoticed by most people. Increasing screenings for oral and pharyngeal cancer is essential to achieving early detection when the disease is most treatable.

**Purpose:**

We tested the effectiveness of a media campaign designed to increase intentions to seek an oral and pharyngeal cancer screening. We further examined whether concern and knowledge of oral and pharyngeal cancer mediated screening intentions.

**Methods:**

Participants in the intervention condition received messages on posters, handheld fans, pamphlets, and magnets displayed on the sides of cars or trucks. Participants in the intervention and comparison conditions (*N* = 1,790) were surveyed prior to and after the intervention.

**Results:**

Intervention participants reported greater intentions to seek free oral and pharyngeal cancer screenings. Concern about oral and pharyngeal cancer partially mediated the effect whereas knowledge did not.

**Conclusions:**

Our media campaign successfully increased screening intentions by heightening concerns.

Oral and pharyngeal cancer is among the most costly and disfiguring of the cancers and the incidence is increasing at selected anatomical sites for both men and women [1, 2]. In addition, compared with other cancers that receive more attention, such as cervical cancer, Hodgkin’s lymphoma, testicular cancer, and thyroid cancer, oral and pharyngeal cancer is particularly lethal, and will claim upwards of 8,000 lives in 2013 [2]. A goal of Healthy People 2020, Health and Human Service’s statement of health goals for Americans, is to increase the proportion of oral and pharyngeal cancer cases diagnosed at earlier, more localized stages [3]. Accomplishing this goal requires implementing health campaigns that increase awareness of oral and pharyngeal cancer and promote greater participation in screenings, especially among people at high risk.

Although oral and pharyngeal cancers are equally common among Whites and Blacks [4], the mortality rate is nearly twice as high among Black men (6.3 per 100,000) than White men (3.7 per 100,000) [5], in part because Blacks are less likely to be diagnosed with oral and pharyngeal cancer at early stages [2]. Oral and pharyngeal cancer is relatively easy [4, 5] and less costly [1] to treat when diagnosed at early stages. However, treatment is more challenging and costly, and mortality more likely, when oral and pharyngeal cancer is diagnosed at late stages. Late-stage diagnosis for oral and pharyngeal cancer is particularly problematic for Blacks living in rural areas who have limited access to healthcare providers who routinely screen for oral and pharyngeal cancer [6].

An obstacle to early diagnosis and to redressing disparities in diagnosis and mortality is lack of public awareness of oral and pharyngeal cancer. Preliminary studies suggest that many people have never heard of oral and pharyngeal cancer and that the low awareness arises in part because healthcare providers, particularly dentists, seem not to discuss oral and pharyngeal cancer with patients [7, 8]. Indeed, oral and pharyngeal cancer as a disease remains unclaimed by both medicine and dentistry. Neither discipline promotes awareness to the extent that is warranted by the survival rates, resulting in public confusion regarding who is responsible for providing routine oral and pharyngeal cancer screening [8].

Reaching the goal of increasing early-stage diagnoses requires health campaigns to increase both public awareness of oral and pharyngeal cancer and the importance the public places on seeking and receiving screenings. We know of only two such health campaigns in the United States. The first used radio and print ads, along with a toll-free hotline, to promote free oral and pharyngeal cancer screenings in and around Detroit, Michigan [9]. Although the campaign motivated more than 1,000 adults to undergo screening, the research lacked measures of the mechanisms responsible for the campaign’s success. The second campaign used billboards and bus-wraps to raise awareness of both oral and pharyngeal cancer and screenings in the intervention city (Jacksonville, Florida) compared with the control city (Tampa, Florida), but was only modestly successful in increasing interest or intentions to seek an oral and pharyngeal cancer screening [10]. Perhaps most important from our perspective is that neither of these studies reported the putative mechanisms responsible for the observed effects.

## Potential Mechanisms

One goal of this study was to identify potential mechanisms by which an oral and pharyngeal cancer media campaign might lead to increased intentions to screen for oral and pharyngeal cancer. The first potential mechanism is knowledge about the disease. Admittedly, although public awareness of oral and pharyngeal cancer is strikingly low, knowledge might seem like an unlikely candidate as a mediator. After all, some 50 years of research suggests that greater knowledge of a health problem is often unrelated to engagement in health behavior [11]. However, some research suggests that increasing knowledge of a health threat can eventuate in the desired health behavior [12]. In addition, a recent focus group study of Blacks in North Central Florida, the target area for our media campaign, revealed that many had never heard of oral and pharyngeal cancer or thought it was unimportant because their physicians never talked about it and because it was not discussed on television or in other media outlets [13]. The low public awareness would suggest that increasing knowledge about the disease is a necessary first step in increasing oral and pharyngeal cancer screening. Presumably, people must know oral and pharyngeal cancer exists, as well as its causes and consequences, before they will take action to address it. Thus, although knowledge and behavior are generally unrelated, they may be related when it comes to seeking screenings. Specifically, because people’s knowledge of oral and pharyngeal cancer is close to zero, a media campaign that increases awareness of oral and pharyngeal cancer and its potential danger may eventuate in a corresponding increase in screening intentions. Thus, we assessed knowledge about oral and pharyngeal cancer and examined the role it might play in influencing screening intentions.

The second potential mechanism was concern about oral and pharyngeal cancer. Health theories and psychological models of persuasion and attitude change specify a variety of mechanisms that govern whether exposure to a message eventuates in changes in thinking and behavior. Some theories, such as the Health Belief Model [14] and the Extended Parallel Processing Model [15], emphasize the role of perceived *threat* in health decision making. Perceived threat reflects both perceived susceptibility and severity of the negative health event. Other theories emphasize the role of *importance* in determining whether people process a message. According to the Elaboration Likelihood Model, people attend to and process messages more deeply when motivation is high [16], and motivation is high when the topic is important (see also the Heuristic and Systemic Model of Information Processing) [17]. Still other theories emphasize the central role of *relevance* in influencing behavior [18]. The common theme underlying these various theoretical mechanisms is *concern* over the health topic or its outcome. Broadly speaking, the more people are concerned about a topic or outcome, the more likely they are to attend to and process relevant messages and, ultimately, engage in health-relevant behaviors such as screening. Accordingly, we examined concern as a potential mediator through which a health media campaign might result in greater intentions to undergo screening for oral and pharyngeal cancer.

## Overview

We tested the effectiveness of a small-media campaign consisting of posters, pamphlets/brochures, handheld fans, and car magnets. The overall aim of the campaign was to promote oral and pharyngeal cancer examinations and to identify the mechanisms by which the media campaign influenced screening intentions. We tailored the media campaign to appeal to rural Black residents by using pictures and facts characterizing the disease among Blacks. Our intent was to increase message salience and the campaign’s effectiveness with Blacks, who are often ignored in health promotion campaigns [19]. We hypothesized that the campaign would influence knowledge and concern about oral and pharyngeal cancer and, ultimately, the residents’ intentions to undergo screening. We further hypothesized that the media campaign would influence screening intentions through concern. Given past research showing that knowledge is sometimes insufficient to produce health changes, we predicted that the media campaign would not influence screening intentions through knowledge.

## Methods

### Media Campaign Intervention

Participants in the intervention community (Alachua, Bradford, Columbia, and Union counties) received an oral and pharyngeal cancer media campaign, whereas participants in the wait-listed comparison community (Gadsden, Jefferson, and Leon counties) did not at that time. We based the media campaign on principles from the Elaboration Likelihood Model [16] and the Extended Parallel Process Model [15] and designed our images and messages to promote in-depth thinking about oral and pharyngeal cancer. According to the Elaboration Likelihood Model, in-depth thinking leads people to form stronger attitudes regarding the relevance of oral and pharyngeal cancer in their lives, which leads to increased effectiveness of the persuasive appeals. The Extended Parallel Process Model proposes that perceptions of susceptibility and severity arouse threat, and that threat, accompanied by feelings of efficacy, motivates risk-reducing and health-enhancing behaviors. We tailored the media campaign to appeal to rural Black residents, who often are ignored in health promotion campaigns. Our goal was to increase message salience and the campaign’s effectiveness with Blacks by including images of Black actors and health facts relevant to Blacks [19].

During the formative phases of our media campaign, focus group participants repeatedly stated a preference for the descriptive term *mouth and throat cancer* instead of *oral pharyngeal cancer*. Most participants felt other names for the cancer were unclear or confusing. As one participant said, “Everybody knows if you say *mouth and throat* where that cancer is going to be.” Following the recommendations of our population of interest, we used the term *mouth and throat cancer* in our media campaign and survey instruments. However, to avoid confusion, we use the term *oral and pharyngeal cancer* throughout this paper.

The formative stage of the campaign included regular, collaborative interactions with community members where we assessed [1] the wording and content of specific information about oral and pharyngeal cancer [2], audience message framing preferences and structure, [3] placement of messages in community locations and businesses, and [4] communication channels such as pamphlets and car magnets. Between May and June of 2009, we conducted five focus groups involving 41 participants (54 % male, 83 % Black) age 51 to 60 to refine message elements. Approximately 12 % of participants lacked a high school diploma. The focus group discussions resulted in 15 prototype messages that presented various messages and that displayed a mix of Black and White actors. We tested the 15 messages in intercept interviews with 149 participants (63 % male, 76 % Black, 7 % White, 17 % declined to provide ethnicity). Participants in the intercept interviews evaluated the 15 messages on a variety of dimensions, including understandability, credibility, likeability, appeal to friends, and overall quality. Nine of the messages were clearly favored by the intercept participants. A second sample of 141 participants recruited at three separate health fairs confirmed the ratings of the nine messages provided by intercept participants.

**Fig. 1:**
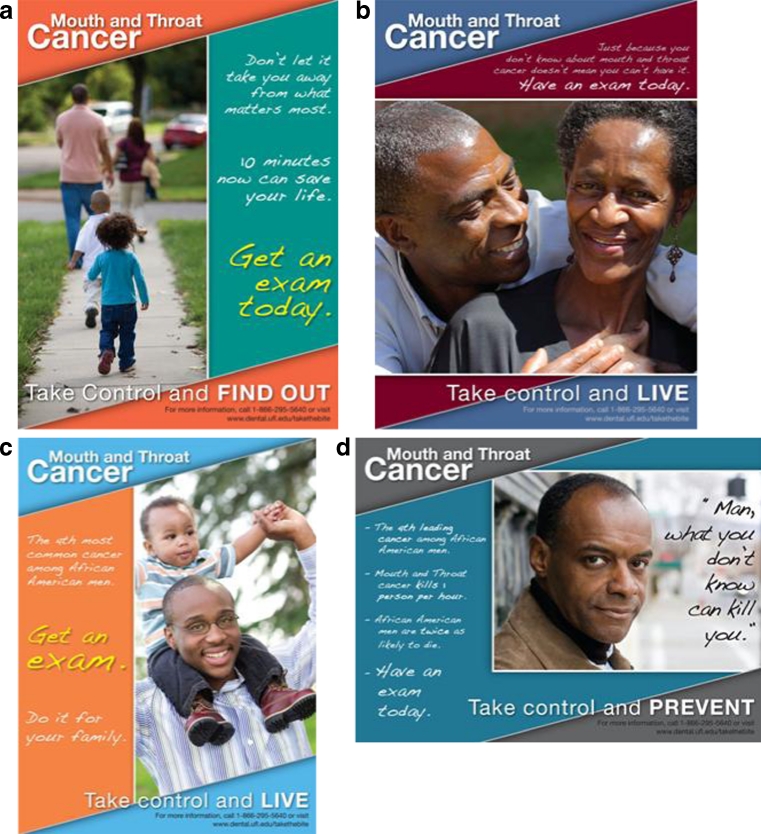
Sample images used in the media campaign

Figure 1 displays four of the nine final messages used in the campaign. The messages included facts and emphasized the seriousness of the disease and Blacks susceptibility to the disease (e.g., “African American men are twice as likely to die as other men,” “What you don’t know can kill you,” and “40,000 people will learn they have mouth and throat cancer this year”). Only one of the nine posters selected by the community participants included White actors. That image was of a racially mixed group of four men (Eddie Van Halen, Sammy Davis Jr., Jim Thorpe, and Babe Ruth) who were all diagnosed with oral and pharyngeal cancer.

The campaign took place in the intervention community from April 2010 to February 2011. Based on suggestions from focus group respondents, we displayed the messages on posters in businesses (primarily minority-owned hair salons, childcare facilities, and medical clinics) and churches in the intervention community. Near the posters were informational brochures that reiterated and expanded the information contained in the posters. Project personnel changed the posters and replenished the brochures approximately every 30 days or more frequently if necessary. We offered a $100 incentive each month for businesses to display the posters. In addition, we provided free, handheld fans to area churches and also distributed these fans at health-related events such as community health fairs. We used the images shown in panels b and c of Fig. 1 on the handheld fans.

Because commonly used venues for media display such as billboards and public transportation vehicles are absent in rural areas, we reproduced an exact image of a poster (see Fig. 1, panel d) on large car magnets that could be affixed to the door panel of vehicles. We asked a non-random sample of 49 residents from the intervention community to affix the car magnets to their personal vehicles for the duration of the campaign. As residents followed their daily routes, message reach increased. Car owners who displayed the magnets received a one-time appreciation payment of $50.

In summary, we used the four images shown in Fig. 1 in the poster displays. We also used panels b and c on the handheld fans, and used the image in panel d on the car magnets.

### Participants and Recruitment

Participants were residents from seven rural, north central Florida counties. For purposes of the pre- and post-surveys, we used 36 census tracts to identify unincorporated areas within the counties that had populations that were greater than 30 % Black. We over-sampled Blacks to ensure adequate representation in our sample. We limited our sampling to homes with landlines to ensure stability in re-contact, obtain clearer communication signals, and increase the likelihood of sampling older adults (who are more likely to use landlines) [20]. We only included people aged 25 or older, and we implemented a within-household respondent selection procedure to maximize participation of older men and help balance representation by gender. We asked for the oldest male in the household, but allowed immediate substitution (of whomever eligible adult was on the phone) if the oldest male was not available [21].

### Telephone Survey Procedure

We used computer-assisted telephone interviews to contact and consent participants and to administer the surveys. Participants received $15 gift cards to Wal-Mart for each of the two surveys they completed, which took approximately 22 min at baseline and 21 min at follow-up. Administration of the baseline survey occurred from November 6, 2009, to March 21, 2010; the follow-up survey administration occurred between March 23 and June 7, 2011. This study was approved by the University of Florida Institutional Review Board.

### Measures

#### Conditional Intention

We measured conditional intention at Time 2 by asking, “If the free exams [for mouth and throat cancer] were offered again, would you come in and be checked?” Participants who responded *No* were coded as 0; participants who responded *Yes* were coded as 1. The rationale for offering a free exam was to remove a frequently identified barrier among these participants, namely cost of examination [7].

#### Perceived Concern

We measured perceived concern about oral and pharyngeal cancer at Time 1 by asking, “How concerned are you about getting mouth or throat cancer in the future?” (1 = *definitely not concerned*; 4 = *very concerned*). At Time 2, we included two additional items using the same four-step response scale: “How concerned are you about your future health overall?” and “Thinking about the important people in your life, how concerned are they about getting mouth or throat cancer in the future?” The three Time 2 items were averaged to form a single index (*α* = .69).

#### Oral and Pharyngeal Cancer Knowledge

We measured knowledge of oral and pharyngeal cancer using 14 true/false items representing possible risk factors for oral and pharyngeal cancer (e.g., *smoking cigarettes*, *pipes*, *or cigars* [true], *eating spicy foods* [false]) and 11 true/false items representing possible warning signs for oral and pharyngeal cancer (e.g., *having trouble swallowing* [true], *sensitive teeth* [false]). We then calculated the percentage of true/false items that participants answered correctly.

#### Message Exposure

We asked participants at Time 2 whether they had seen messages about mouth and throat cancer on posters in businesses, on handheld fans, in pamphlets or flyers, or on the sides of cars or trucks. We created an index of exposure that ranged from 0 (*saw none of these four types of messages*) to 4 (*saw all types of messages*). Our rationale for this index was that seeing multiple novel delivery methods for the messages should be more influential than seeing a single type repeatedly. Participants reported seeing the pamphlet (32.8 %) most commonly, followed by the poster (28.6 %), handheld fans (4.15 %), and car magnets (3.20 %).

#### Demographics

We collected demographic information in the Time 1 survey. We mean-centered age and coded race so that 1 = *Black* and 0 = *White*, and gender so that 0 = *female* and 1 = *male*. We assessed financial security using two items [22, 23]. The first item asked participants, “Which of these statements best describes your present financial status?” 1 = *I really can*’*t make ends meet*, 2 = *I manage to get by*, 3 = *I have enough to manage plus some extra*, and 4 = *Money is not a problem*; *I can buy about whatever I want*. The second item asked, “If you were faced with an unexpected $500 medical bill that was not covered by insurance, how would you best describe your situation?” 1 = *Not able to pay the bill*, 2 = *Able to pay*, *but with difficulty*, and 3 = *Able to pay comfortably*. A continuous financial security scale (range = 0 to 2, with two indicating highest financial security) was created from a weighted average of the two items. We coded education into six categories: 1 = *eighth grade or less*, 2 = *some high school*, *but did not graduate*, 3 = *high school graduate or GED*, 4 = *some college or two*-*year degree*, 5 = *four*-*year college graduate*, and 6 = *more than a four*-*year degree*. Finally, we coded communities so that 1 = *comparison community* and 0 = *intervention community*.

### Data Analysis

**Fig. 2:**
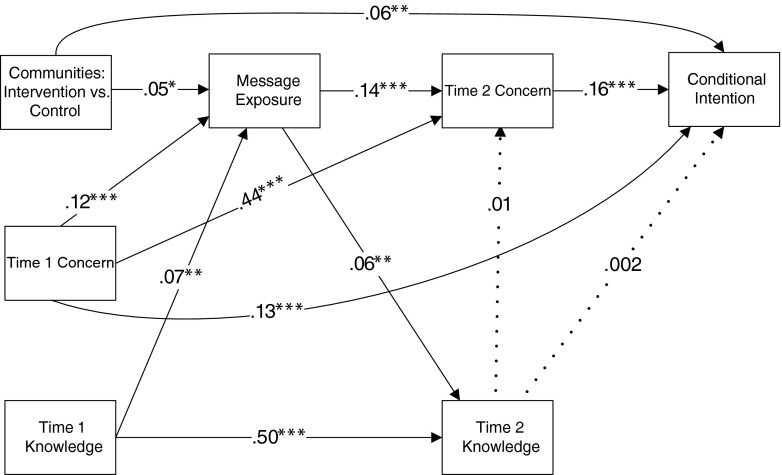
The full model. Note: *p < .05; **p < .01; ***p < .001. *Dashed lines* indicate non-significant paths

We developed a path model to test the effects of the media campaign on conditional intention to get screened (Fig. 2). The exogenous variables included media campaign (intervention vs. comparison community), Time 1 concern, and Time 1 knowledge. The endogenous variables included message exposure, Time 2 concern, Time 2 knowledge, and conditional intention.

In path analysis, an endogenous variable can be an outcome variable in one relationship and a predictor variable in another relationship. These variables are called mediators. In our study, we assessed mediation by ordering the exogenous variables based on the logic of our intervention and performing regression analysis in a structural and systematic way. Specifically, we assumed that our media campaign would influence message exposure (dependent variable), which distinguishes participants in the two communities (independent variable). Message exposure (independent variable) should in turn influence Time 2 concern and knowledge (dependent variables). Finally, Time 2 concern and knowledge (independent variables) should influence our outcome measure, conditional intention (dependent variable).

Age, gender, race, education, and financial security were controlled in the path model. We performed our path analysis using methods described in Menard Chapter 8 [24]. We conducted all analyses using SAS version 9.3 (SAS Institute, Cary, NC).

## Results

### Description of Sample

Of the approximately 16,000 phone numbers dialed, 2,605 people agreed to participate and completed the baseline (Time 1) survey and 1,881 (72.2 %) of these participants completed the follow-up (Time 2) survey. Each phone number was called up to 10 times (Time 1) or 25 times (Time 2), between 9 am and 9 pm weekdays and weekends. Those who were lost to attrition were more likely to be younger and Black, compared with those who completed both surveys. Retention was similar for the intervention (75.0 %) vs. comparison (74.5 %) counties. We eliminated participants who did not report their race as either Black or White, resulting in a final sample of 1,790 participants (57 % women) who provided complete data. The sample was 26 % Black (*n* = 473) and 74 % White (*n* = 1,317), with 46 % (*n* = 828) residing in the intervention counties (Alachua, Bradford, Columbia, and Union counties) and 54 % (*n* = 962) residing in the wait-listed comparison counties (Gadsden, Jefferson, and Leon counties). Various health organizations offered free screenings on a limited basis in both the intervention and comparison counties in the 5 years preceding the follow-up survey. Hereafter we refer to the intervention counties as the intervention community and the comparison counties as the comparison community. Table 1 provides descriptive statistics for the intervention and comparison communities and the overall sample. The average age of participants at baseline was 57.4 years (SD = 14.7). More women than men from both communities participated in the survey (56.1 % from the intervention community and 55.9 % from the comparison community). For the total sample, 24.6 % had completed high school or had a GED and 30.5 % had some college. The mean response on our index of financial security was 1.17, suggesting that the overall financial security may be low (scale ranged from 0–2). Overall, 64.2 % of the participants (69.1 % in the intervention community and 59.5 % in the comparison community) reported conditional intentions to get screened.

**Table 1:** Characteristics of the intervention and comparison communities

		Intervention community (*n* = 962)	Comparison community (*n* = 828)	*p* value	*r* value
Age		57.0 (15.5)	57.6 (13.9)	.42	−.02
Gender				.77	−.01
	Male	43.9 %	44.1 %		
	Female	56.1 %	55.9 %		
Race				.01	−.06
	White	77.5 %	72.2 %		
	Black	22.5 %	27.8 %		
Education				.001	−.17
	8th grade or less	2.4 %	1.6 %		
	Some HS	7.5 %	4.3 %		
	Completed HS or GED	28.5 %	20.8 %		
	Some college	35.3 %	25.8 %		
	College graduate	13.0 %	22.2 %		
	Post-graduate	13.3 %	25.3 %		
Financial security (range 0–2)		1.10 (0.6)	1.24 (0.6)	.001	−.09
Conditional intention				.001	.08
	Yes	69.1 %	59.5 %		
	No	30.9 %	40.5 %		

All percentages are survey sampling weighted

*HS* high school, *GED* general equivalency degree (or diploma)

### Preliminary Analyses

Table 2 provides the zero-order correlations between all variables and reveals that the comparison and intervention communities were matched in terms of age and gender (*r*s < .02, ns). Of note, a greater proportion of White participants lived in the intervention community than in the comparison community (*r* = −.06, *p* < .01). Compared with the participants in the comparison community, participants in the intervention community reported lower levels of education (*r* = −.17, *p* < .001) and lower financial security (*r* = −.09, *p* < .001). In addition, participants in the intervention community reported more exposure to the oral and pharyngeal cancer messages (*r* = .05, *p* < .05) and greater conditional intention (*r* = .08, *p* < .001). Greater knowledge corresponded with less concern at both Time 1 and Time 2 (*r*s ≥ −.13, *p*s < .0001). In addition, greater knowledge corresponded with lower screening intention (*r*s ≥ −.07, *p*s < .05), whereas greater concern corresponded with greater screening intention (*r*s ≥ .28, *p*s < .0001).

**Table 2:** Correlations

	Age	Gender	Race	Education	Financial security	Community	Message exposure	Time 1 concern	Time 2 concern	Time 1 knowledge	Time 2 knowledge
Gender	−.00										
Race	−.10***	.06*									
Education	−.07**	.02	−.19***								
Financial security	.15***	.09***	−.25***	.39***							
Community	−.02	−.01	−.06**	−.17***	−.09***						
Message exposure	−.06*	.01	.22***	−.17***	−.10***	.05*					
Time 1 concern	.00	.06*	.23***	−.23***	−.25***	.02	.18***				
Time 2 concern	.02	.05*	.27***	−.28***	−.26***	.03	.25***	.53***			
Time 1 knowledge	−.15***	−.07**	−.25***	.24***	.16***	−.03	−.02	−.13***	−.15***		
Time 2 knowledge	−.18***	−.07**	−.22***	.25***	.17***	−.04	.02	−.15***	−.15***	.53***	
Conditional Intention	−.03	−.00	.27***	−.23***	−.27***	.08***	.12***	.28***	.32***	−.07**	−.08***

*N* = 1,790

*p < .05; **p < .01; ***p < .001

### Test of a Mediation Model

As shown in Fig. 2, we observed a significant path from community (intervention vs. comparison) to conditional intention (*β* = .06, *p* < .01), indicating that participants reported greater conditional screening intentions in the intervention condition than in the comparison condition. We also observed an indirect path between community and conditional intention. Specifically, community predicted message exposure such that participants reported greater message exposure in the intervention community than in the comparison community (*β* = .05, *p* < .05). Greater message exposure in turn predicted greater concern at Time 2 (*β* = .14, *p* = <.0001). Importantly, because we controlled for concern at Time 1, we can interpret message exposure as predicting a *change* in concern from Time 1 to Time 2. Finally, greater concern at Time 2 predicted greater conditional screening intention (*β* = .16, *p* < .0001).

**Fig. 3:**
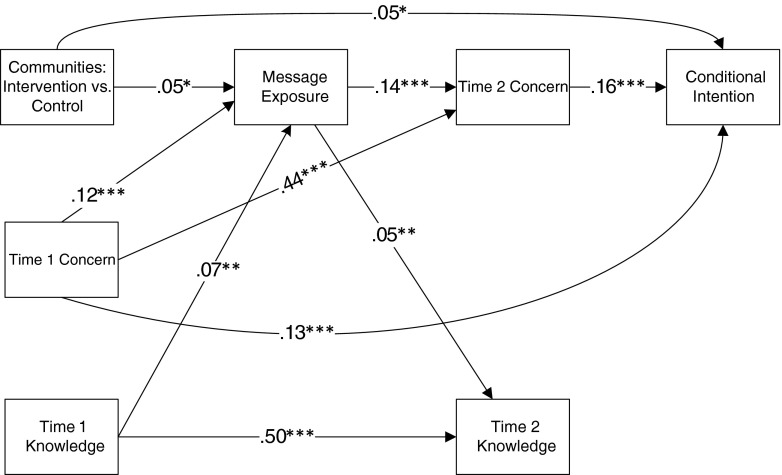
The trimmed model. Note: *p < .05; **p < .01; ***p < .001

We also observed a significant path from message exposure to Time 2 knowledge (*β* = .06, *p* < .01). However, Time 2 knowledge did not predict either Time 2 concern (*β* = .01, ns) or conditional intention (β = .002, ns). Thus, Time 2 knowledge played no role in screening intention and did not mediate the effect of community on conditional intention. Finally, as evident in Fig. 3, trimming the model of non-significant paths did not negatively affect the fit of the model. Both the full and the trimmed models provide good fit to the data. For both models, the comparative fit index is 1 and the root mean square error of approximation is smaller than .001. Additional calculations showed that the direct effect of community on conditional intentions was .05 and the indirect effect (community to message exposure to Time 2 concern to conditional intent) was .001 (.05 × .14 × .16).

**Fig. 4:**
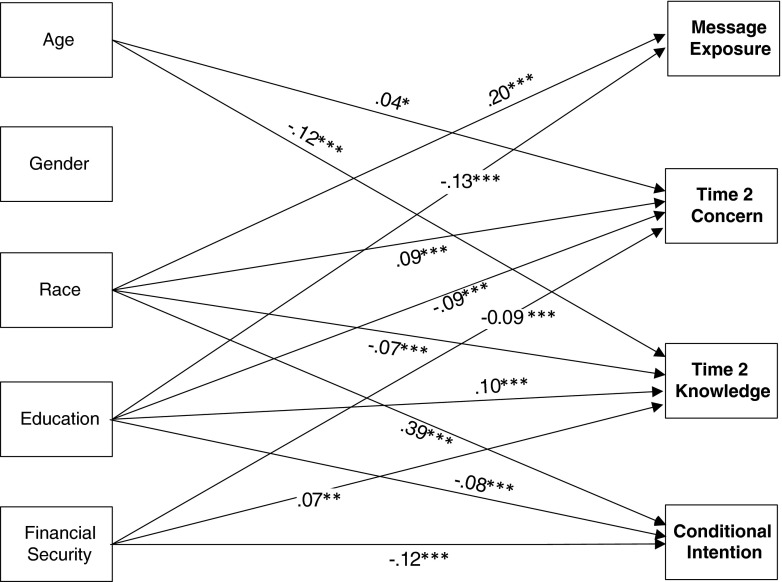
Control and endogenous variables. Note: *p < .05; **p < .01; ***p < .001

Although we controlled for age, gender, race, education, and financial security in our path model, the correlation matrix suggests several important relationships between the demographic, mediator, and outcome variables. Figure 4 depicts the significant standardized coefficients between the control variables and the endogenous variables. The path coefficients represent the effect of the demographic variables on the endogenous variables after controlling for all other variables (including community, Time 1 knowledge, and Time 1 concern). Age corresponded with Time 2 concern and Time 2 knowledge, such that older adults reported greater concern (*β* = .04, *p* < .05), but less knowledge (*β* = −.12, *p* < .0001) than did younger adults. We also observed significant paths from race and education to each of the endogenous variables. Regarding race, compared with White participants, Black participants reported greater message exposure (*β* = .20, *p* < .0001), greater concern (*β* = .09, *p* < .0001), less knowledge (*β* = −.07, *p* < .0001), and greater intentions to get screened (*β* = .39, *p* < .0001). Regarding education, greater education corresponded with less message exposure (*β* = −.13, *p* < .0001), less concern (*β* = −.09, *p* < .0001), greater knowledge (*β* = .10, *p* < .0001), and lower intentions to get screened (*β* = −.08, *p* < .0001). We also observed significant paths between financial security and three of the endogenous variables. Specifically, greater financial security corresponded with less concern (*β* = −.09, *p* < .0001), greater knowledge (*β* = .07, *p* < .01), and lower intentions to get screened (*β* = −.12, *p* < .0001). Finally, gender was unrelated to any of the endogenous variables.

We note that American actor-director Michael Douglas announced that he was diagnosed with oral and pharyngeal cancer during the media campaign of our study. We examined whether this announcement influenced our findings by analyzing whether participants at Time 2 differed before vs. after the announcement in responses to message exposure as well as concern, knowledge, and conditional intention. Analysis revealed no significant effects, all *F*s < 1, all *p*s > .39, suggesting that the Douglas announcement did not influence our findings.

### Discussion

Previous studies of the effect of media campaigns on oral and pharyngeal cancer screenings have shown some positive outcomes with urban populations but have not examined potential mechanisms responsible for that success. The current study extends prior research on oral and pharyngeal cancer media campaigns in two ways. First, we tailored our campaign to rural Black residents using focus group methodology. Second, we used path modeling to identify potential mechanisms through which the media campaign influenced intentions to get screened.

Our media campaign successfully increased conditional screening intentions. The increase in intentions stemmed partly from the media campaign raising oral and pharyngeal cancer-related concerns. As we noted at the outset, concern over a health topic or outcome represents a common mechanism of many theories of persuasion and attitude change, and also with many health theories. For instance, the Health Belief Model [14] and the Extended Parallel Processing Model [15] conceptualize concern in terms of perceived threat, which reflects both perceived susceptibility and severity of a negative health event. These theories propose that people are more likely to pursue health-related actions, such as getting screened, when threat is high. Likewise, the Elaboration Likelihood Model, which featured prominently in the development of our messages, proposes that when a concern about an issue is high (i.e., topic is important), people are more motivated to attend to issue-relevant messages and process them more deeply [16]. From our perspective, concern addresses the fundamental idea that people must be concerned about a health event—they care about it and see it as important and relevant to their lives—before they will take health relevant action. Thus, people must be concerned about oral and pharyngeal cancer before they are willing to undergo screening. Our media campaign increased screening intentions in part by elevating concerns.

Consistent with prior research [11, 25], knowledge did not influence screening intentions. Although participants in the media campaign community showed greater knowledge of oral and pharyngeal cancer than did participants in the comparison community, their greater knowledge did not translate into greater intentions to get screened. Our findings are a reminder that media campaigns relying solely on information-only materials are unlikely to produce health changes.

In addition to our primary findings regarding concern and knowledge and their relationship to screening intentions, our study revealed several important findings relevant to our demographic variables. First, Black participants reported greater exposure to the messages than did White participants, indicating that our strategy of tailoring the message to Black participants was successful. Second and more importantly, participants who were Black, who were less educated, and who were less financially secure reported at Time 2 greater concern about oral and pharyngeal cancer and intentions to get screened. All of these effects emerged after controlling for Time 1 knowledge and concern. Given that these groups are arguably at greater risk for negative consequences because of later-stage diagnosis, these findings are encouraging. They suggest that an opportunity for free screening would eventuate in greater screening uptake and ultimately identification of oral and pharyngeal cancer at earlier stages when it is most easily treated.

Black participants in our study reported greater message exposure, and we believe as a consequence, greater concern about oral and pharyngeal cancer at Time 2 and ultimately greater screening intentions than did White participants. This finding is not surprising given that our posters featured images of Black actors and were displayed mostly in Black businesses and as magnets on cars driven by Black residents presumably in mostly Black neighborhoods. Yet, we also found that education correlated negatively with message exposure and subsequently, with concern at Time 2 and with screening intentions even after controlling for race and financial security. We suspect that education functioned in our study as a proxy for oral cancer risk factors (e.g., smoking and heavy alcohol use) and for having regular dental care. Accordingly, participants with few risk factors and regular dental care were naturally less concerned about getting oral cancer and less interested in obtaining a free screening if we offered the opportunity.

### Strengths and Limitations

Our study has several strengths. Foremost, our media campaign breaks novel ground. It is among the first oral and pharyngeal cancer interventions developed specifically for rural residents. Second, our large sample size, method of recruitment, and careful implementation of the campaign in the intervention community make us confident in our effect and in the generality of our findings to adult residents in rural Florida community. Third, our effects were unaffected by the announcement by Michael Douglas, which received wide coverage by the popular press.

These strengths aside, our media campaign had several limitations. First, we drew our sample from rural residents in north Florida, and it remains to be seen whether the findings generalize to other regions of the country. Second, many of our measures were single items, raising concerns regarding item reliability. Third, although intentions often predict behavior [26], we did not assess behavior in this study. Fourth, because perceived concern and conditional intentions were both assessed during the same wave (T2), we acknowledge the possibility that conditional intentions influenced concern rather than the reverse. Fifth, we presented detailed facts and information about oral and pharyngeal cancer in the brochures/pamphlets, but presented only limited facts and information in the messages, thus weakening a putative knowledge gain effect. Finally, we examined intentions to get a free screening if one were available. Responses among these rural residents may have differed had we asked about screening that was not free or about getting treated if diagnosed with oral and pharyngeal cancer, particularly among residents with financial constraints.

In spite of these limitations, our study demonstrated success in reaching rural minority individuals using small, relatively inexpensive media. Individuals in the intervention community reported seeing the messages and reported positive intentions to get screened. As hypothesized, changes in concern partially mediated this relationship. Importantly, the direct effect remained significant even with the mediators in the model, suggesting the possible presence of other mechanisms influencing conditional screening intentions among our participants. It is also possible that our mediators were imprecisely measured and that we need more valid and reliable measures of the components of concern such as importance, relevance, perceived severity, and perceived threat. We also acknowledge that other factors, such as response and self-efficacy and normative beliefs, can influence intentions. Future research should explore these factors in assessing campaigns to promote oral and pharyngeal cancer screening.

## Conclusion

The Institutes of Medicine [27] recently announced a critical need to increase access to oral health care for vulnerable and underserved populations and a need for research to address oral health disparities. Our study responds to these needs and reports a pathway on which to build future health messages tailored for rural minority residents.
